# Laparoscopic partial gastrectomy for gastric calcifying fibrous tumor: a case report and literature review

**DOI:** 10.1097/RC9.0000000000000230

**Published:** 2026-02-18

**Authors:** Emiri Sugiyama, Itaru Yasufuku, Yu Tajima Jessie, Yoshihiro Tanaka, Chiemi Saigo, Nobuhisa Matsuhashi

**Affiliations:** a Department of Gastroenterological and Pediatric Surgery, Gifu University Graduate School of Medicine, Gifu, Japan; b Department of Pathology and Translational Research, Gifu University Graduate School of Medicine, Gifu, Japan

**Keywords:** calcifying fibrous tumor, stomach, submucosal tumor

## Abstract

**Introduction:**

Calcifying fibrous tumor (CFT) is a rare benign lesion that occasionally arises in the gastrointestinal tract. Gastric CFT is extremely uncommon and often mimics gastrointestinal stromal tumor (GIST), making preoperative diagnosis challenging.

**Presentation of Case:**

We report a 46-year-old man with a gastric submucosal tumor detected during a medical checkup. During 3 years of follow-up, the tumor increased in size from 20 mm to 30 mm. Endoscopic ultrasonography revealed a hyperechoic lesion located medial to the muscularis propria, and abdominal computed tomography showed a calcified submucosal mass with contrast enhancement. Fine-needle aspiration biopsy failed to provide a definitive diagnosis, and malignant potential, including GIST, could not be excluded. Therefore, laparoscopic partial gastrectomy was performed. Histopathological examination revealed dense collagen, psammomatous calcification, spindle cells, and lymphoplasmacytic infiltrates, consistent with CFT. The final pathological tumor size was 15 mm. Immunohistochemistry showed positivity for vimentin and negativity for c-kit, DOG1, SMA, and CD34. The postoperative course was uneventful, and there has been no recurrence observed during 2.5 years of follow-up.

**Discussion:**

Gastric CFT is rare, with only 22 cases reported worldwide since 2014. Most cases of gastric CFT were managed by endoscopic resection, while laparoscopic surgery has rarely been reported. Radiologic and endoscopic findings may help differentiate CFT from GIST, but definitive diagnosis requires histopathology.

**Conclusion:**

Laparoscopic resection is a safe and effective treatment option when gastric CFT cannot be preoperatively distinguished from GIST. Awareness of this rare entity may prevent overtreatment and guide optimal surgical decision-making.

## Introduction

Calcifying fibrous tumor (CFT) is a rare benign lesion that typically arises in the limbs, trunk, neck, or deep soft tissues, but is extremely uncommon in the gastric wall[1]. Calcifying fibrous submucosal tumors (SMTs) are difficult to differentiate from other SMTs, such as lipomas, neuroendocrine tumors, and gastrointestinal stromal tumors (GISTs). Therefore, there are only a few case reports of gastric CFT^[^2–5^]^. CFT in the gastrointestinal tract occurs predominantly in middle-aged adults, with a slight female predominance. The most frequently reported sites of CFT are the small bowel and colon, followed by the stomach and appendix[6]. Gastric CFTs are often relatively large at the time of diagnosis and require surgical resection. We report a rare case of a gastric CFT that was successfully treated by laparoscopic partial gastrectomy.

HIGHLIGHTS

- Calcifying fibrous tumor (CFT) of the stomach is an extremely rare benign lesion.
- It is difficult to preoperatively distinguish a gastric CFT from a stromal tumor.
- A gastric CFT was successfully resected via laparoscopic partial gastrectomy.
- Histology shows collagen, calcification, and lymphoplasmacytic infiltration.
- Minimally invasive resection is a safe treatment option for gastric CFT.

This case report has been reported in line with the SCARE 2025 checklist[7].

## Presentation of case

A 46-year-old man was diagnosed with a gastric SMT during a medical checkup. The lesion was monitored for 3 years, during which esophagogastroduodenoscopy revealed interval growth, prompting referral to our hospital.

The patient was asymptomatic on presentation. His history included an anxiety disorder and appendectomy at 8 years of age He was not taking any regular medications and had no known allergies. There was no relevant family history, and he neither smoked nor drank alcohol. Physical examination was unremarkable, and routine blood and biochemical tests were within normal ranges. Serum concentrations of tumor markers were normal (carcinoembryonic antigen: 2.2 ng/mL; carbohydrate antigen 19-9: 30.5 U/mL).

**Figure 1.**
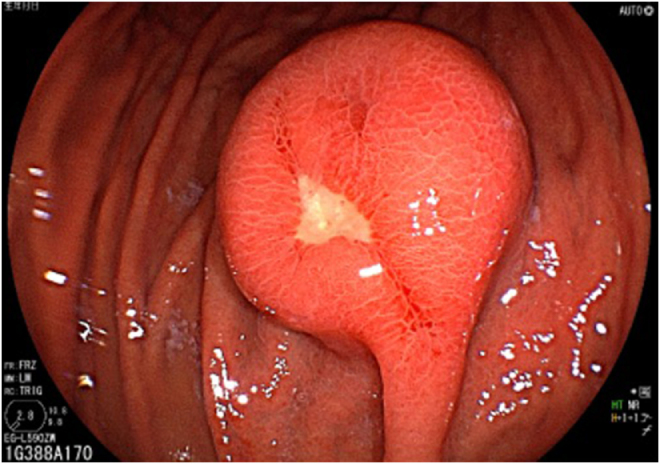
Endoscopic view of the gastric submucosal mass in this report. The mass has a smooth surface with a central ulcer.

**Figure 2.**
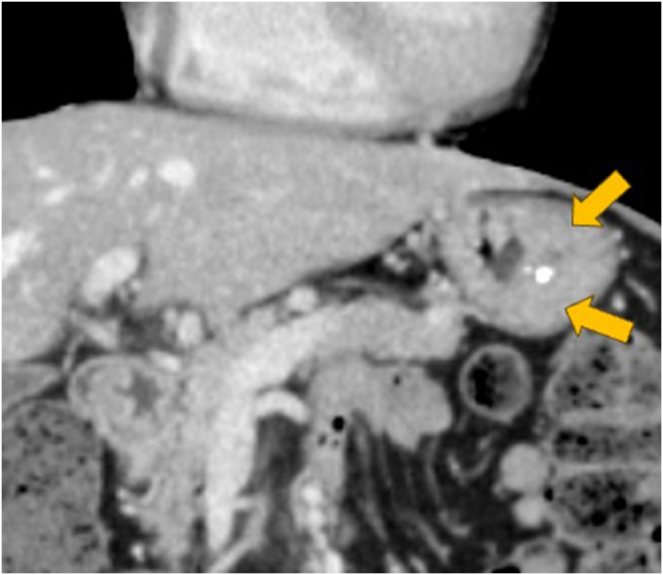
Abdominal computed tomography images show a submucosal tumor with calcification (left) and incremental contrast enhancement (right).

Endoscopy revealed a circular SMT in the greater curvature of the upper gastric body that measured 30 mm at the time of referral (compared with 20 mm when first measured 3 years earlier) and had a smooth surface and central ulcer (Fig. 1). Endoscopic ultrasonography (EUS) showed a homogeneous hyperechoic mass located medial to the muscularis propria (fourth layer of the gastric wall) (Supplemental Digital Content Figure 1, available at: http://links.lww.com/IJSCR/A11). These EUS features – homogeneous hyperechogenicity and lack of continuity with the muscularis propria – are atypical for GIST and, in retrospect, suggested the possibility of CFT, although they were not diagnostic preoperatively. Abdominal computed tomography demonstrated a 16-mm submucosal mass with calcification and incremental contrast enhancement (Fig. 2). No lymphadenopathy or distant metastases were observed.

**Figure 3.**
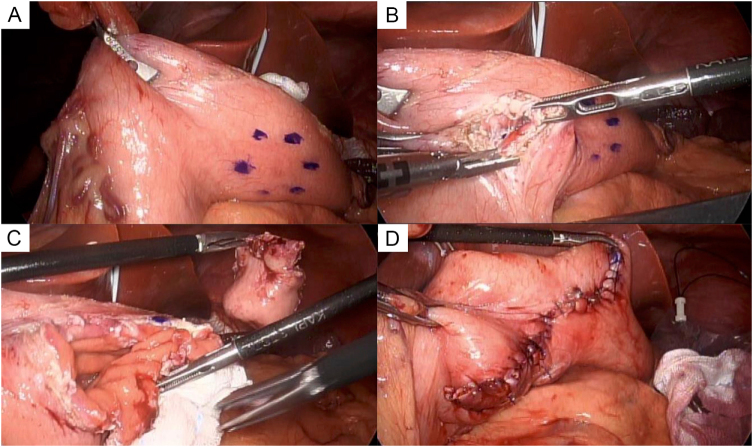
Laparoscopic partial gastrectomy. (A) The omentum is divided from the stomach. (B) The gastric wall excision begins. (C) The tumor is excised. (D) The gastric wall defect is closed with 4-0 absorbable barbed suture.

EUS-guided fine-needle aspiration was attempted but failed to yield a definitive diagnosis. Because GIST could not be excluded, laparoscopic partial gastrectomy was performed. No special preoperative optimization was required other than standard fasting. Perioperative management included standard intravenous antibiotics and venous thromboembolism prophylaxis. Intraoperatively, the tumor had no serosal exposure or peritoneal dissemination. The greater omentum was divided laparoscopically, and the tumor was resected with wide margins. The gastric wall was closed manually with 4-0 absorbable barbed suture. The operation time was 141 minutes with negligible blood loss (Fig. 3A–D). The procedure was performed by a gastrointestinal surgeon with 8 years of surgical training at our institution, which has extensive experience in gastrointestinal and minimally invasive surgery. There was no deviation from the initial management plan, and the laparoscopic approach was successfully completed as intended.

**Figure 4.**
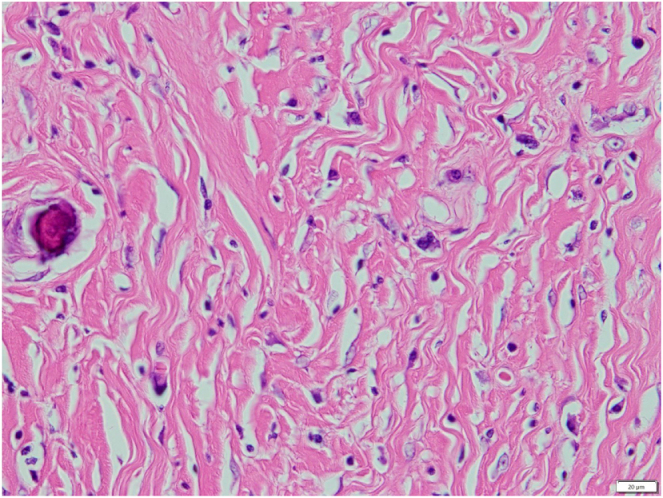
Microscopic findings. Microscopically, the tumor consists of dense collagenous tissue containing sparse spindle cells, psammomatous calcification, and multifocal lymphoplasmacytic infiltrates. (A) Hematoxylin and eosin stain, original magnification × 10. (B) Hematoxylin and eosin stain, original magnification × 200.

**Figure 5.**
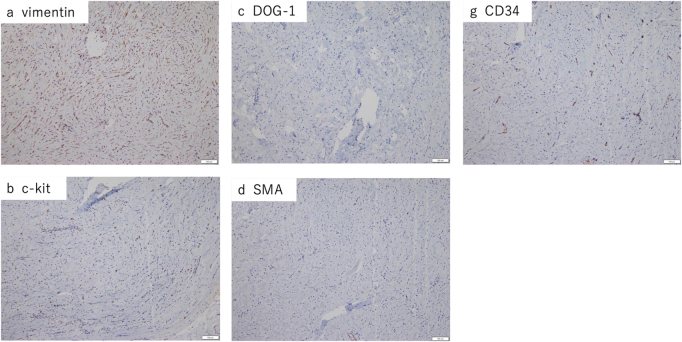
Immunohistochemical staining showing vimentin positivity and negativity for c-kit, DOG1, SMA, and CD34 (×200).

Macroscopically, the tumor was firm, well-circumscribed, and white, with a diameter of 15 mm (Supplemental Digital Content Figure 2, available at: http://links.lww.com/IJSCR/A11). Microscopically, it consisted of dense hyalinized collagen, sparse spindle cells, psammomatous calcification, and multifocal lymphoplasmacytic infiltrates (Fig. 4). Immunohistochemically, the tumor was positive for vimentin and negative for c-kit, DOG1, SMA, desmin, S-100, CD34, EMA, and GLUT-1. The Ki-67 index was 1% (Fig. 5). These findings were diagnostic for CFT. The surgical margins were negative.

### Follow-up

The patient was followed up at our outpatient clinic with the operating surgeon. Follow-up visits were scheduled every 3–6 months, with clinical examination and imaging studies including endoscopy and CT. At 2.5 years after surgery, the patient remains recurrence-free. No specific long-term surveillance or postoperative restrictions were required.

Adherence and compliance: The patient adhered well to postoperative instructions and tolerated the recovery without difficulty. No specific restrictions or compliance issues were noted.

### Outcomes

The expected outcome was complete resection with no recurrence, in line with previous reports describing the excellent prognosis of gastric CFT. The attained outcome was consistent with expectations, as the patient remained recurrence-free at 2.5 years after surgery.

### Complications

Perioperative prophylaxis included intravenous antibiotics and venous thromboembolism prevention. No intraoperative or postoperative complications occurred (Clavien–Dindo classification: Grade 0). The patient was discharged within the expected timeframe and experienced no morbidity within 30 days or during long-term follow-up.

### Timeline of the case report

2021/04 – gastric submucosal tumor was detected during health screening endoscopy.

2021/05–2022/10 – regular endoscopic follow-up demonstrated gradual tumor enlargement.

2022/10 – CT and EUS suggested a GIST.

2022/11 – Laparoscopic partial gastrectomy was performed.

2023/05 – 6-month postoperative follow-up showed no recurrence.

2025/05 – 2.5-year follow-up confirmed no recurrence.

## Discussion

CFT is a rare benign tumor first described in the extremities of children in 1988[8]. While most reported cases originate from subcutaneous or deep soft tissues, visceral CFTs – including those of gastric origin – are much less frequent^[^2–5^]^. A review of 157 cases reported that the most common sites of CFT are the stomach (18%), pleura (9.9%), small intestine (8.7%), peritoneum (6.8%), neck (6.2%), mesentery (5%), and mediastinum (5%)[9]. A slight female predominance has been reported, with a bimodal age distribution peaking in early childhood and in the third decade of life[9]. Although an association between CFT and IgG4-related disease has been suggested due to IgG4-positive plasma cells, the relationship remains unclear[10].

Radiologically, gastric CFT often appears as a hyperattenuating mass on computed tomography owing to intratumoral calcification, although isoattenuated and enhanced tumors have also been described[5]. It is crucial to differentiate CFT from GIST, as calcification is rare in GIST. On EUS, CFT typically appears as a small, hard, well-demarcated mass with expansile growth, absence of continuity with the muscularis propria, and heterogeneous high echogenicity. In contrast, GISTs usually show irregular margins, muscle layer continuity, and hypoechoic heterogeneity[11]. In our case, the lack of continuity with the muscularis propria supported the diagnosis of CFT, but it was not possible to obtain a definitive diagnosis preoperatively. Although the lesion appeared approximately 30 mm on endoscopy, this macroscopic estimation included the elevated mucosa and submucosal tissue. The final pathological tumor diameter was 15 mm, which explains the discrepancy commonly observed in submucosal tumors.

To date, no gastric CFT has been definitively diagnosed by EUS-guided fine-needle aspiration alone, highlighting the challenges in preoperative diagnosis. Histological evaluation of spindle-shaped cells, calcification, and collagen deposition is essential, and immunohistochemistry is required to exclude other spindle cell neoplasms, such as GIST. Histologically, CFT is characterized by hypocellular hyalinized collagen, psammomatous or dystrophic calcification, spindle fibroblasts, and lymphoplasmacytic infiltrates with lymphoid aggregates[9]. Immunohistochemically, CFT is typically positive for vimentin and factor XIIIa and negative for c-kit, SMA, desmin, actin, S-100, and ALK-1, with occasional CD34 positivity^[^9,11^]^. Our findings were consistent with these criteria. A PubMed search identified 22 gastric CFTs reported between 2014 and 2024, including our case^[^5,11–17^]^. The clinicopathological features of these reported cases are summarized in Table 1. Patient age ranged from 22 to 71 years (median, 45 years), with a female predominance (14/22). Tumor size ranged from 10 to 29 mm (median, 16.5 mm). Most lesions were located in the gastric body. Eighteen patients underwent endoscopic resection, while only four underwent surgery. To our knowledge, this is the first reported case of gastric CFT treated with a purely laparoscopic partial gastrectomy. None of the reported patients developed recurrence following either endoscopic or surgical treatment.

**Table 1:** Cases of CFT reported between 2014 and 2024.

Characteristic	N = 22
Age, years	22–71 (45)
Sex	
Male	8
Female	14
Size, mm (mean)	10–29 (16.5)
Location	
Gastric fundus	2
Upper gastric body	3
Gastric body	15
Gastric antrum	1
NR	1
Clinical manifestations	
Epigastric pain, abdominal pain	6
Abdominal discomfort	1
Incidental finding	8
NR	7
Surgery	
ESD	13
ESE	5
LECS	2
PGR	1
LPGR	1
Recurrence	
None	18
NR	4

ESD: endoscopic submucosal dissection; ESE: endoscopic submucosal excavation; LECS: laparoscopic and endoscopic cooperative surgery; LPGR: laparoscopic partial gastrectomy; NR: not recorded; PGR: partial gastrectomy.

Although CFT is benign and there have been no reported cases of metastasis or disease-related death, local recurrence has occasionally been described^[^1,18^]^. Therefore, wide excision is recommended when surgical resection is performed. For gastric lesions, endoscopic resection may be considered; however, when GIST cannot be excluded, laparoscopic surgery is appropriate and safe[19]. Our case highlights the role of laparoscopic resection in achieving definitive diagnosis and curative treatment when preoperative investigations are diagnostically inconclusive.

## Strengths and limitations

This case highlights the feasibility and safety of laparoscopic partial gastrectomy for gastric CFT, an extremely rare lesion that is often difficult to differentiate preoperatively from GIST. The strength of this report lies in the detailed radiological and pathological findings, which may aid in future differential diagnosis. However, the limitation is the relatively short follow-up period and the fact that it is a single case, which restricts the generalizability of the findings.

## Patient perspective

When I was told that the tumor in my stomach might be malignant, I was very anxious. After receiving thorough explanations from my doctors, I decided to undergo surgery. I am relieved that the operation was successful, and I hope that sharing my experience will be helpful for other patients.

## Conclusion

Gastric CFT is an uncommon benign tumor that radiologically and endoscopically mimics GIST. We report a rare case of gastric CFT successfully treated by laparoscopic partial gastrectomy. Recognition of CFT in the differential diagnosis of calcified gastric SMTs is important to avoid overtreatment and to guide appropriate surgical management.

## Data Availability

Data sharing is not applicable to this article as no datasets were generated or analyzed.

## Conference presentation

This case was presented at the International Gastric Cancer Congress (IGCC) 2025.

## Other journal submission

This case is not under consideration at any other journal.
